# Advanced Time-Frequency Methods for ECG Waves Recognition

**DOI:** 10.3390/diagnostics13020308

**Published:** 2023-01-13

**Authors:** Ala’a Zyout, Hiam Alquran, Wan Azani Mustafa, Ali Mohammad Alqudah

**Affiliations:** 1 Department of Biomedical Systems and Informatics Engineering, Yarmouk University, Irbid 21163, Jordan; 2 Faculty of Electrical Engineering & Technology, Campus Pauh Putra, Universiti Malaysia Perlis, Arau 02000, Perlis, Malaysia; 3 Advanced Computing, Centre of Excellence (CoE), University Malaysia Perlis (UniMAP), Arau 02000, Perlis, Malaysia

**Keywords:** ECG, iris-spectrogram, scalogram, CNN, ResNet101, ShuffleNet, heart rhythm

## Abstract

ECG wave recognition is one of the new topics where only one of the ECG beat waves (P-QRS-T) was used to detect heart diseases. Normal, tachycardia, and bradycardia heart rhythm are hard to detect using either time-domain or frequency-domain features solely, and a time-frequency analysis is required to extract representative features. This paper studies the performance of two different spectrum representations, iris-spectrogram and scalogram, for different ECG beat waves in terms of recognition of normal, tachycardia, and bradycardia classes. These two different spectra are then sent to two different deep convolutional neural networks (CNN), i.e., Resnet101 and ShuffleNet, for deep feature extraction and classification. The results show that the best accuracy for detection of beats rhythm was using ResNet101 and scalogram of T-wave with an accuracy of 98.3%, while accuracy was 94.4% for detection using iris-spectrogram using also ResNet101 and QRS-Wave. Finally, based on these results we note that using deep features from time-frequency representation using one wave of ECG beat we can accurately detect basic rhythms such as normal, tachycardia, and bradycardia.

## 1. Introduction

The heart is a muscle that pumps blood throughout the body, and contracts rhythmically. The atrial sine node, which functions as a natural pacemaker, initiates this contraction, which then spreads to the rest of the muscle. There is a pattern to the way that this electrical pulse spreads [1]. This action causes fluctuations in the skin’s surface’s electrical potential by producing electric currents on the body’s surface. Electrodes and proper tools can be used to record or measure these signals, known as an electrocardiogram (ECG) [2].

**Figure 1:**
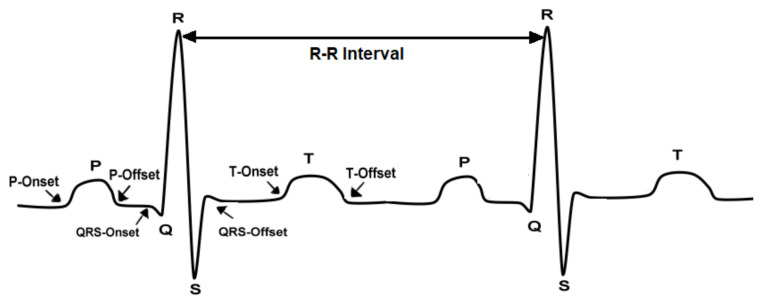
ECG signal components show the onset and offset of each wave.

An ECG signal is composed of three major components explained in Figure 1 [3]: P-wave; QRS complex, which contains three waves, i.e., Q, R, and S; and the T-wave [4]. The P-wave is a small flexure wave indicating atrial depolarization, ventricular depolarization is represented by the QRS complex, and the T-wave is indicative of ventricular repolarization (atrial repolarization is hidden by the large QRS complex) [5]. The amplitudes and frequencies of these waves are shown in Table 1 below [4,6].

When there is no disease or abnormality in the waveform of the ECG signal, the heart’s regular rhythm is known as a normal sinus rhythm (NSR). Typically, the heart rate of NSRs ranges from 60 to 100 beats per minute. The breathing cycle causes a small change in the R-R interval’s regularity. Sinus tachycardia is the name for the rhythm when the heart rate rises above 100 beats per minute and the R-R interval decreases. This is the heart’s normal response to the need for increased blood circulation; it is not an arrhythmia. However, overly rapid heartbeats result in incomplete filling of the ventricles before contraction, which lowers pumping effectiveness and negatively impacts perfusion. Bradycardia, which occurs when the heartbeat is extremely slow, can have a significant negative impact on important organs and the heart rate drops down to 60 beats per minute, and the R-R interval increases [7].

The paper is organized as follows: Section 2 provides details about the state-of-the-art-related works. Section 3 presents an explanation of the used dataset, the preprocessing and segmentation of the ECG, iris spectrogram, and scalogram. Section 4 shows the results, including the performance of the proposed CNN classifiers, and the discussion about the proposed method results. Finally, Section 5 represents the conclusion of the work.

## 2. Literature Review

Classification of normal and arrhythmia-associated ECG is an important goal to achieve better detection and proper identification of various cardiovascular diseases (CVDs). However, the small amplitude and duration of the ECG arrhythmia can make it difficult to classify. With the rise of deep learning techniques, several recent studies have used very deep networks for ECG classification. Here, we will attempt to detail the latest related works using time-frequency methods for ECG classification.

Rashed Al-Mahfuz et al. proposed a novel ECG beat classifier using a customized VGG16-based Convolution Neural Network (CNN) with two advanced time-frequency representation techniques, Continuous Wavelet Transforms (CWT) and Hilbert-Huang transform (HHT), to identify the best time-frequency representation of ECG beats. The proposed adopted CNN with CWT scalogram achieved 100% classification accuracies on MIT-BIH arrhythmia database for 2–4 classes and 99.90% for 5 classes, and the CWT scalogram outperformed the HHT spectrum in all the cases [8].

Swain et al. introduced an automated identification of myocardial infarction (MI) using a modified Stockwell transform (MST)-based time-frequency analysis and a phase information distribution pattern method. Both healthy and MI ECG signals are collected from the PTB diagnostic ECG database with 12 lead ECG signals; the results of the proposed method can detect the MI successfully with an accuracy, sensitivity, and specificity of 99.93%, 99.97%, and 99.30% respectively [9]. Additionally, Lekhal et al. introduced an ECG beat classifier system based on features observed in time–frequency analysis using a variant of the Stockwell transform, and then the SVM with asymmetric costs (AS3VM) was applied for assessment of the feature performance. The proposed method has been evaluated on the MIT-BIT arrhythmia database, using four types: normal beats (N), left and right bundle branch blocks (L and R), and premature ventricular contractions (V). The obtained results show accuracies of 99.35%, 98.73%,98.57%, and 99.44% respectively, for N, L, R, and V beats [10].

However, a suitable method for telemedicine systems provided by Kayikcioglu et al. to classify ST segment using time-frequency distribution based on features from multi-lead ECG signals of four-class and tested them on three different databases, MIT-BIH Arrhythmia database, European ST-T database, and Long-Term ST database. The weighted k-NN algorithm achieved the best average performance with an accuracy of 94.23%, a sensitivity of 95.72%, and a specificity of 98.15% using the Choi–Williams time-frequency distribution features, in addition to the other classification algorithms SVM and Ensemble [11].

Kłosowski et al. proposed an effective method for ECG classification using the deep neural long-short-term memory (LSTM) network and feature extraction consists of converting the ECG signal into a series of spectral images using short-term Fourier transformation. Then, the images were converted using Fourier transform again to two signals, which include instantaneous frequency and spectral entropy, which are used to train the LSTM network [12].

In 2021, Wang et al. provided a simple and accurate method, which can be used as a clinical auxiliary diagnostic tool, and is an automatic ECG classification method based on Continuous Wavelet Transform (CWT) to obtain different time-frequency components and Convolutional Neural Network (CNN) to extract features from the 2D scalogram composed of the time-frequency components. The method achieved an accuracy of 98.74%, a sensitivity of 67.47%, an F1-score of 68.76%, which compared with existing methods is increased by 4.75~16.85%, and a positive predictive value of 70.75% [13].

In the same year, Hussein et al. presented a novel method to extract ST and PR features from the Choi–Williams time-frequency distribution proposed for myocardial ischemia identification. With the use of these extracted features, a multi-class SVM classifier is trained to detect unknown circumstances and assess whether they are ischemic or normal. Improved detection performance is the result of using multi-lead ECG for classification and 1 min intervals rather than beats or frames. The proposed strategy produced a final result that had an overall accuracy, sensitivity, and specificity of 99.09%, 99.49%, and 98.44%, respectively [14].

Furthermore, in 2022, Alqudah et al. published a paper in which they present a method that is efficient, simple, fast, and deployable on mobile devices. A deep learning methodology was developed to detect up to 17 classes of cardiac arrhythmia based on analyzing a single ECG beat and calculating the iris spectrogram to feed the convolutional neural network. The results show that the proposed methodology has an overall recognition accuracy of 99.13% ± 0.25, 98.223% ± 0.85, and 97.494% ± 1.26 for 13, 15, and 17 arrhythmia classes, respectively. The training/testing is performed using tenfold cross-validation [15].

Faraget et al. provided a short-time Fourier Transform (STFT) Convolutional Neural Network (CNN) model for ECG classification in real-time at the edge. To extract the spectrogram from the input ECG signal, they developed an STFT-based 1D convolutional (Conv1D) layer and then reshaped it into a 2D heat-map image to feed the 2D convolutional (Conv2D) neural network (CNN) for classification. The proposed classifier achieved 99.1% accuracy and a 95% F1-score at the edge with a maximum model size of 90 KB, an average inference time of 9 ms, and a maximum memory usage of 12 MB [16].

This study aims to propose a comparison between two different advanced time-frequency methods, i.e., iris spectrogram and scalogram, to categorize the previous types of ECG, i.e., Normal, Tachycardia, and Bradycardia, using deep learning with Resnet101 and ShuffleNet convolutional neural networks.

## 3. Materials and Methods

**Figure 2:**
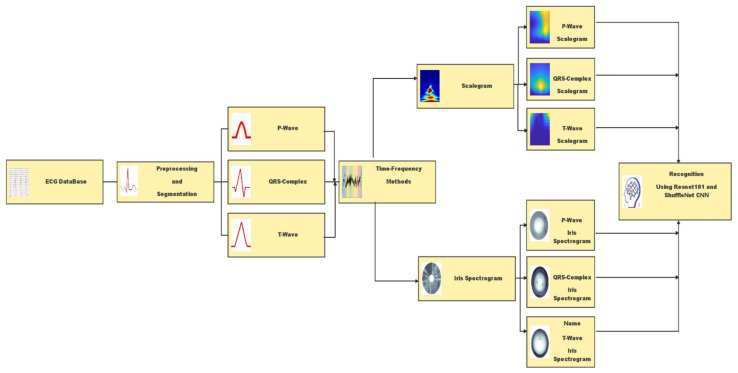
Block diagram of the proposed methodology.

The methodology of this research can be concentrated on the design and implementation of the procedure steps to achieve the overall approach. The first step is preparing ECG data with different types to be ready for the system implementation. The implemented approach for ECG wave recognition is passed into three main steps, as shown in Figure 2, the Preprocessing and Segmentation process, Time-Frequency methods, and diagnosis and Recognition using Deep Learning.

### 3.1. Dataset

ECG-ID Database is used in this study for normal ECG type; the database contains 310 ECG recordings, obtained from 90 persons, and was created and contributed by Tatiana Lugovaya, who used it in her master’s thesis [17]. Additionally, the Challenge 2015 Training Sets is used for tachycardia and a bradycardia type; the training set contains 750 recordings for five ECG categories. However, two types were selected [18]. The length of each ECG signal segment is 10 s. The applied segmentation methodology (discussed in the next section) results in 316 normal beats, 138 Bradycardia, and 326 Tachycardia images for all methods, with a total of 780 images.

### 3.2. ECG Preprocessing and Segmentation

The main objective of this processing is to distinguish the P, QRS, and T waves and detect the characteristic points P-Onset, P-Offset, QRS-Onset, QRS-Offset, and T-Onset, T-Offset for each cycle. After identifying the ECG waves, their respective amplitudes are measured concerning the baseline. Before analysis, an ECG signal is typically the first bandpass filtered using several frequency ranges. Bandpass filtering is widely used to remove low- and high-frequency noise components, baseline wander muscle noise, and power line interference [19]. The frequency range used is 0.5–40 Hz [20,21].

**Figure 3:**
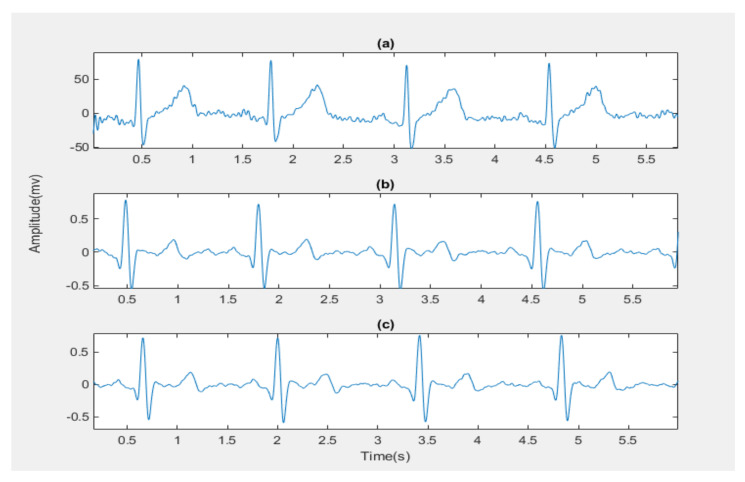
The preprocessing stages for normal ECG signal for example: (**a**) The Original ECG signal; (**b**) The Preprocessed ECG signal; (**c**) The ECG signal starting with the P-wave and ending with the T-wave.

For de-noising and baseline wandering removal, different types of wavelet transforms were usually applied to ECG signals. Advanced signal processing methods, such as the stationary wavelet transform de-noising technique, should be employed to eliminate various noise types that contain muscle artefacts and electrode moving artefacts [22]. Baseline wanders removal and de-noising were achieved by multiresolution wavelet transform [23]. In this study, the noisy signal was decomposed into nine levels by using the Daubechies wavelet db8. The de-noised signal was recovered by taking the inverse discrete wavelet transform of the resulting coefficients. The next step in the preprocessing stage used is amplitude normalization, which is optional but useful for visually comparing data from various patients and datasets [24]. To ensure the signal starts with P-wave and ends with T-wave, find the peaks of the R-wave. The onset of the P-wave for the signal is the half peak-to-peak distance between the two first peaks and the offset of the T-wave for the signal is also the half peak-to-peak distance between the two last peaks. Figure 3 below show the preprocessing stages for all ECG data.

**Figure 4:**
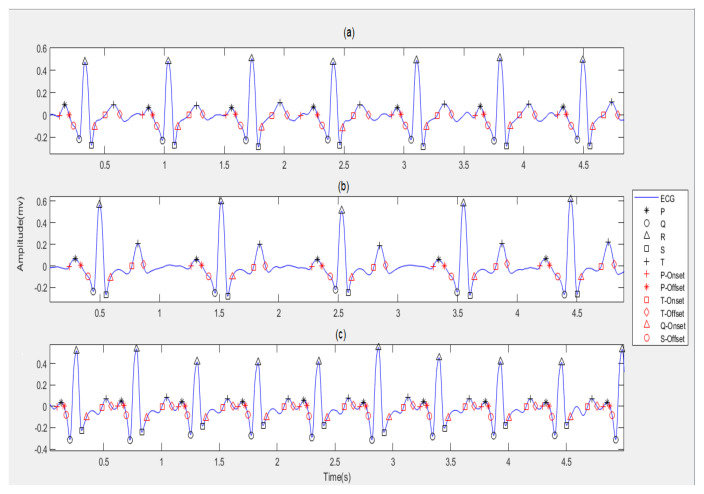
The peaks and intervals detection for ECG types: (**a**) The Normal ECG; (**b**) The Bradycardia ECG; (**c**) The Tachycardia ECG.

**Figure 5:**
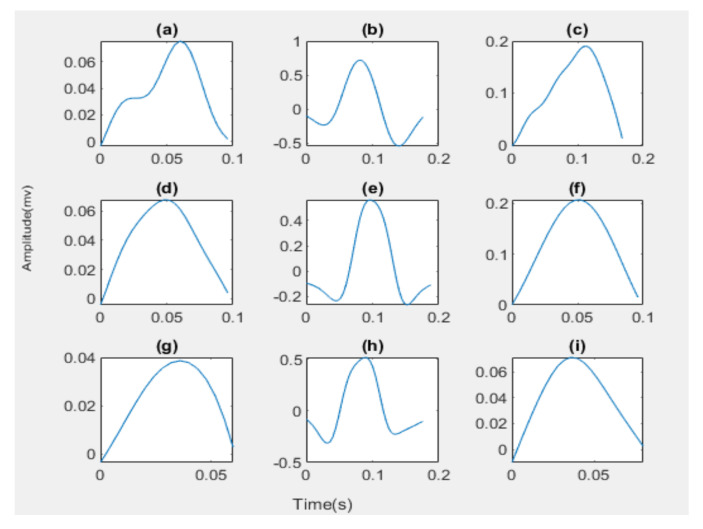
The wave segmentation for ECG types: (**a**) The Normal P-wave; (**b**) The Normal QRS-waves; (**c**) The Normal T-wave; (**d**) The Bradycardia P-wave; (**e**) The Bradycardia QRS-waves; (**f**) The Bradycardia T-wave; (**g**) The Tachycardia P-wave; (**h**) The Tachycardia QRS-waves; (**i**) The Tachycardia T-wave.

In this work, we have adopted the QRS-waves detection algorithm, which is developed by Faruk U, who proposed a new thresholding method on the Pan–Tompkins algorithm [25] and found the maximum peaks in the left and the right of the R-wave within a window to detect the P-wave and the T-wave of the same beat. Furthermore, in this work, we have depended on the zero crossing to detect the onset and offset of each wave; the P-wave and T-wave onset and offset was detected by beginning from the waves’ peaks and by searching backwards and forward, respectively, to find zero crossing, which is the onset and offset of the two waves, respectively. From the P-wave offset to Q-wave, a forward search is carried out in this window to find the last zero crossing point. This point is the QRS onset. To find the QRS offset, we take a window from the S-wave to T-wave onset and search forward in this window to find the first zero crossing. Figure 4 and Figure 5 illustrates the process of segmentation.

For segmenting, ECG waves are considered at an extracted onset and offset of each wave separately from the ECG signal for all ECG types as shown in Figure 5. A total of 320 for normal, 140 for bradycardia, and 325 for tachycardia P-waves, QRS-waves, and T-waves are extracted.

### 3.3. Time-Frequency Representations

Time-frequency representations define the frequency content of a signal as a function of time [26]. Time-frequency analysis and representation are used in the fields of signal and image processing, data analysis, measurements, acoustics and vibration, machinery diagnosis, seismology, etc. for the analysis of signals and data, fundamental frequency detection, instantaneous frequency determination, etc. [27]. Two of the most commonly used time–frequency analysis tools include the short-time Fourier transform (STFT) and continuous wavelets transform (CWT) [28].

STFT is a series of Fourier transforms of a windowed signal. When a signal’s frequency components change over time, the STFT gives time-localized frequency information, whereas the conventional Fourier transform provides frequency information averaged across the whole signal time interval. The spectrogram, which is an intensity representation of STFT magnitude over time, is frequently used to visualize STFT [29]. The STFT is given by:

(1) $$X\left(n,\omega \right)=\sum_{m=-\infty }^{\infty }\omega \left[n-m\right]x\left[m\right]e^{-j\omega n}$$

*m* is a “dummy” time argument whereas n represents the location of the short segment of the original time function as it is obtained by the window $\omega \left[n-m\right]$ which moves along the m-axis according to the value of n.

Wavelet decomposition uses a scale rather than a frequency to translate a signal onto a time-scale plane. The time-frequency plane of the STFT is the same as this, and each scale of the time-scale plane corresponds to a certain frequency range of the time-frequency plane. When comparing the wavelet with the Fourier transform, the wavelet decomposes the signal into shifted or scaled shapes from a mother wavelet, whereas the Fourier analysis decomposes the signal into sinusoids of various frequencies [30].

The CWT is the sum of the signal $x\left(t\right)\;$ multiplied by shifted and scaled shapes from a mother wavelet $\varphi \left(t\right)$ [30]:

(2) $$CWT\left(scale,position\right)=\int_{-\infty }^{+\infty }x\left(t\right)*\varphi \left(scale,position,t\right)dt\;$$

At this time, frequency–time analysis allows us to know how the signal is distributed with frequency and phase, so complex signals can be expressed concisely and analyzed easily. However, by visually representing signals at various scales and various frequencies through CWT, hidden features can be seen in the time-frequency domain [30].

#### 3.3.1. Irisgram ECG Representation

**Figure 6:**
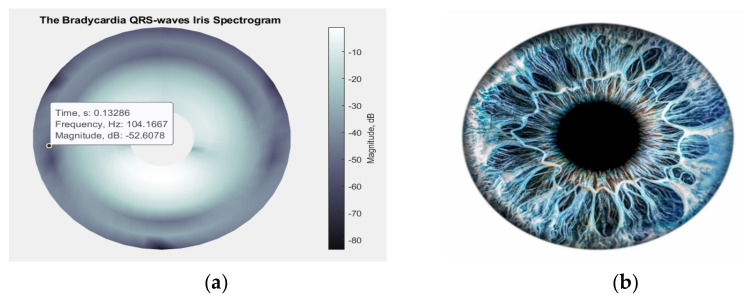
(**a**) An irisgram of QRS-waves; (**b**) Human eye iris.

In 2018, H. Zhivomirov proposed an innovative method for visualizing the outcomes of time-frequency analysis called an “iris-spectrogram” or “irisgram” for its resemblance to a human iris, as shown in Figure 6. The irisgram is a circular representation of the traditional spectrogram in which the signal strength is indicated axially with color and time increases circumferentially in a clockwise direction. To pinpoint the precise location of a given point in the time-frequency domain, one must utilize a Data Cursor tool because the circular design of the irisgram does not permit annotations to be placed on the time and frequency axes [27].

Times and frequencies values converted into the polar coordinates to generate an irisgram, using the following Equations (3)–(5), are applied [31]:

(3) $$\theta =\left\{-\pi :\frac{2\pi }{T-1}:\pi \right\},\;where\;T\;is\;the\;length\;of\;time\;vector.$$

(4) $$\rho =\frac{\max\left(f\right)}{3}+f,\;where\;f\;is\;the\;frequency\;vector.$$

(5) X=ρ×cosθ , and Y=ρ×sinθ.

Then, compute the spectrogram of power spectral density (PSD) and convert to amplitude spectrum in dB within the range −120 dB to 120 dB, which are the values of the Z plane [32]. Using the (surf) function, the values of X and Y are plotted against Z to create a 3D surface plot. This sort of plot contains solid edge colors and face colors, and the color of the surface varies according to the heights that Z specifies. This function is used to plot the values in matrix Z as heights (weights) above a grid in the X-Y plane [15].

**Figure 7:**
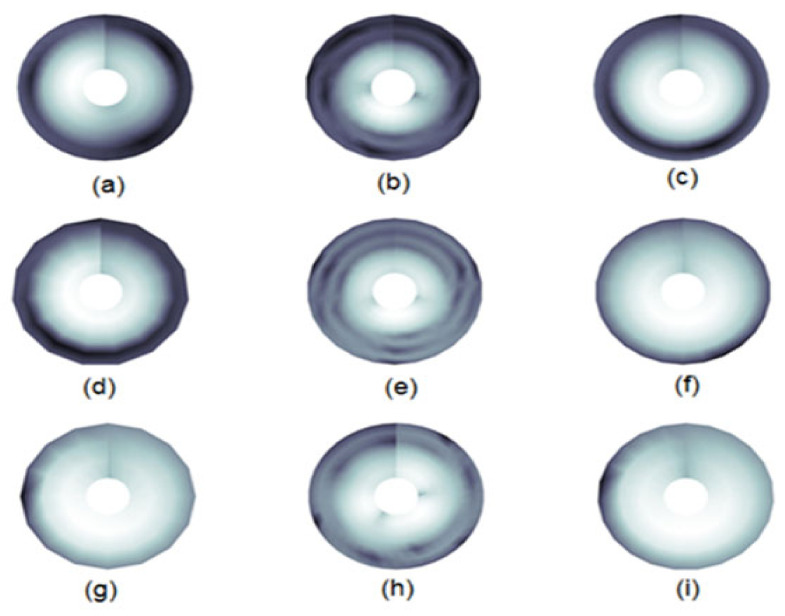
The irisgrams for ECG types: (**a**) The Normal P-wave irisgram; (**b**) The Normal QRS-waves irisgram; (**c**) The Normal T-wave irisgram; (**d**) The Bradycardia P-wave irisgram; (**e**) The Bradycardia QRS-waves irisgram; (**f**) The Bradycardia T-wave irisgram; (**g**) The Tachycardia P-wave irisgram; (**h**) The Tachycardia QRS-waves irisgram; (**i**) The Tachycardia T-wave irisgram.

In this research, for all types of segmented ECG waves, the irisgram representation has been generated using the function *irisgram*, accessed in MATLAB^®^ Central File Exchange [32], and the resulting images are stored with their relevant classes, as shown in Figure 7. Then, the deep learning models will be applied to the stored images, as will be shown next.

#### 3.3.2. Scalogram ECG Representation

The scalogram (SG) is a time-frequency representation of the signal constructed by a wavelet transformation, where coefficient values at specific time-frequency locations can be indicated by color [33]. The SG of x is defined by the following equation:

(6) $$S\left(x\right)=∥W_{a}x\left(b\right)∥\sqrt{\int_{-\infty }^{+\infty }x\left(t\right)\varphi (\frac{t-B}{A}})da\;$$

**Figure 8:**
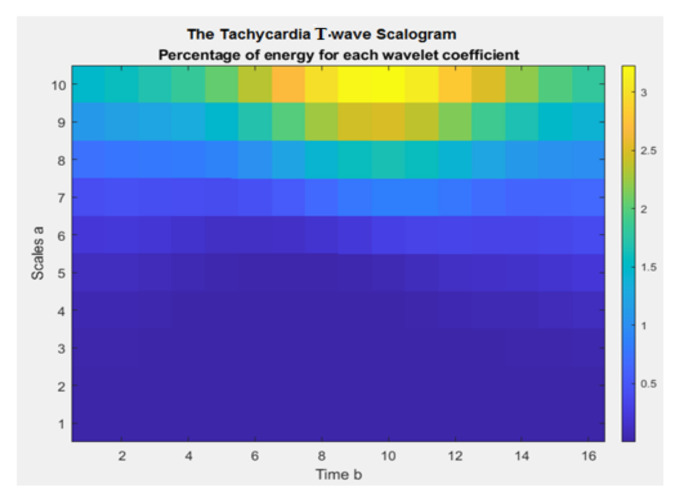
The Tachycardia T-wave SG.

Which represents the energy of $W_{a}x\left(b\right)$ at the scale A. B defines the translation of the mother wavelet $\varphi \left(t\right)$. The SG enables the detection of the signal’s most representative scales (or frequencies), or those that contribute the most to the signal’s overall energy. By integrating (4) between these values, we may define the appropriate windowed SG if we are only interested in a specific time window (t_0_; t_1_). The three axes are time (x), scales (y), and coefficient value (z) [34,35]. The ECG wave SG sample is shown in Figure 8.

**Figure 9:**
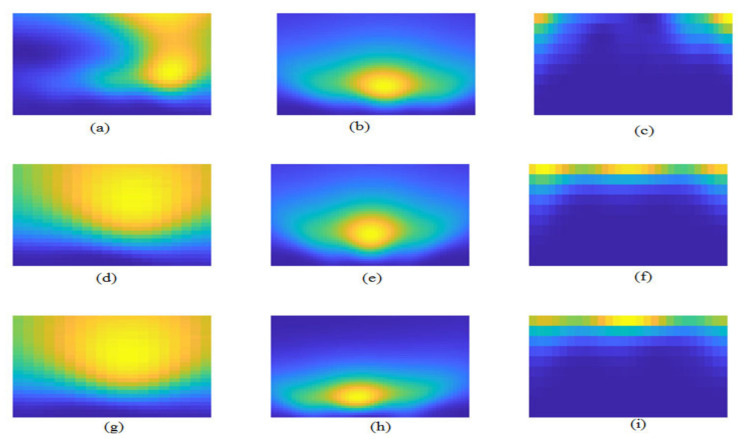
The Scalogram for ECG types: (**a**) The Normal P-wave SG; (**b**) The Normal QRS-waves SG; (**c**) The Normal T-wave SG; (**d**) The Bradycardia P-wave SG; (**e**) The Bradycardia QRS-waves SG; (**f**) The Bradycardia T-wave SG; (**g**) The Tachycardia P-wave SG; (**h**) The Tachycardia QRS-waves SG; (**i**) The Tachycardia T-wave SG.

Additionally, in this research, for all types of segmented ECG waves, the SG representation has been generated using the function *wscalogram*, in MATLAB^®^, with different scaling in each wave, as shown in Table 1, and the resulting images are stored with their relevant classes, as shown in Figure 9. Then, the deep learning models will be applied to the stored images, as will be shown next.

### 3.4. Deep Learning

Usually, deep learning models need a large dataset to train and achieve robust results. Therefore, many researchers have started to employ transfer learning techniques to tune the pre-trained deep learning structures to perform the intended task.

The first convolutional neural network is the residual neural network, which is distinguished by its residual block property. This feature enhances the performance of classification by overcoming the problems of vanishing or exploding gradients due to deep learning layers. ResNet allows forming a skipping connection which enables activating a layer to further layers by skipping some layers in between. There are various versions of ResNet, such as ResNet-18, -34, -50, and -101. These versions are based on the number of deep layers. The architecture ResNet is stacking of such residual blocks. The input size of these networks is 224 × 224 × 3 [36].

The second convolutional neural network is ShuffleNet, which is one of the most effective networks utilized in mobile applications. To obtain a high accuracy level, ShuffleNet performs two types of convolutions: point-wise group convolution and channel convolution, which makes its performance efficient and fast. It consists of a stacking of ShuffleNet blocks, each one consisting of two grouped convolutional layers, channel shuffle layer, in addition to depth-wise convolutional layers. The output from each block maps using the ReLU layer. The designed input layer is compatible with image size 224 × 224 × 3 [37,38].

## 4. Results

The resulting images were utilized to build deep learning models either using ResNet101 or ShuffleNet. ECG signal is segmented into three waves P, QRS, and T. Each segment proceeds with irisgram and scalogram, separately. For each wave, there are two generated colored images; one for scalogram and the other for irisgram. The labeled data are recognized based on ECG diagnosis, normal, bradycardia, or tachycardia. For each class, six datasets are achieved; three ECG segments for each category in both signals’ representations, i.e., irisgrams and scalograms. The classification is performed using two pre-trained deep-learning structures ResNet and ShuffleNet. The resulting representation images are divided into 70% training and 30% testing. The corresponding sections demonstrate the analysis of the results.

### 4.1. Irisgram Representation

#### 4.1.1. ResNet

The iris image classification is executed using pre-trained ResNet101 structures, and the corresponding matrices illustrate its performance. The first one shows the performance of the irisgram of the P-waves, and the second one illustrates the capabilities of the irisgram of the T-waves. The third represents the performance of the irisgram of the QRS waves.

**Figure 10:**
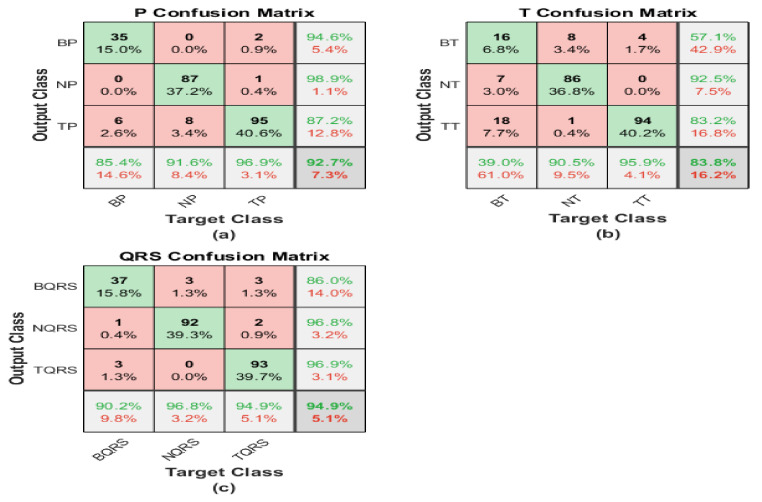
Confusion matrices of ECG irisgram using ResNet101: (**a**) P-waves irisgram; (**b**) T-waves irisgram; (**c**) QRS waves irisgram.

For the irisgram P-wave, as shown in Figure 10a, the sensitivity of bradycardia is 85.4%, where 35 out of 41 cases are classified correctly. The precision of bradycardia is 94.6%. Meanwhile, 87 segments were discriminated from 95 P-waves for normal subjects, with a sensitivity of 91.6% and a positive predictive value of 98.9%. A true positive rate of tachycardia is the highest value at 98.9%. On the other hand, its precision is the lowest because 14 cases were misrecognized as tachycardia. The overall accuracy is 92.7% for all classes.

The confusion matrix describes the outputs of T-waves. The results are not promising. Seven segments are misclassified as healthy and 18 cases of bradycardia are misclassified as tachycardia. Therefore, the sensitivity is too low for bradycardia cases, at 39%. Moreover, 14 cases from other classes are misclassified as bradycardia by the worst precision of 57%. The performance of ResNet is better regarding normal class recognition. Nine samples are misclassified, which is one as bradycardia and the rest as tachycardia, with a sensitivity of 90.5% and a misclassification rate of 9.5%. Regarding discrimination between tachycardia and normal classes, there are seven classes of bradycardia classified as normal and the precision is 92.5%. Tachycardia’s sensitivity reaches 95.9%, and the misclassification rate is 4.1%. Furthermore, 19 cases of bradycardia cases are recognized as tachycardia. Therefore, the precision was reduced to 82.2%. The overall accuracy is the lowest, and it does not exceed 83.3%.

For the QRS wave irisgram, results show an improvement in discrimination between three classes in terms of sensitivity, accuracy, and precision. In the bradycardia class, 37 cases are distinguished correctly from 41 and six misclassified samples as bradycardia. That is why the precision reduced to 86% from what it was in the P-waves case. Recall and PPV are the highest for healthy segments by almost 97% for both performance terms. Tachycardia obtains a high level of precision using the QRS-waves, in which just three samples from the whole data were misclassified as tachycardia, and the sensitivity is 94.9%. The overall accuracy of the proposed approach regarding QRS-waves irisgram is 94.9%.

#### 4.1.2. ShuffleNet

The irisgram images are proceed using ShuffleNet. The process is started by splitting the dataset into 70% training and 30% tests. That operation is executed on each ECG segment. The following confusion matrices characterize the performance of the test phase of the whole database.

**Figure 11:**
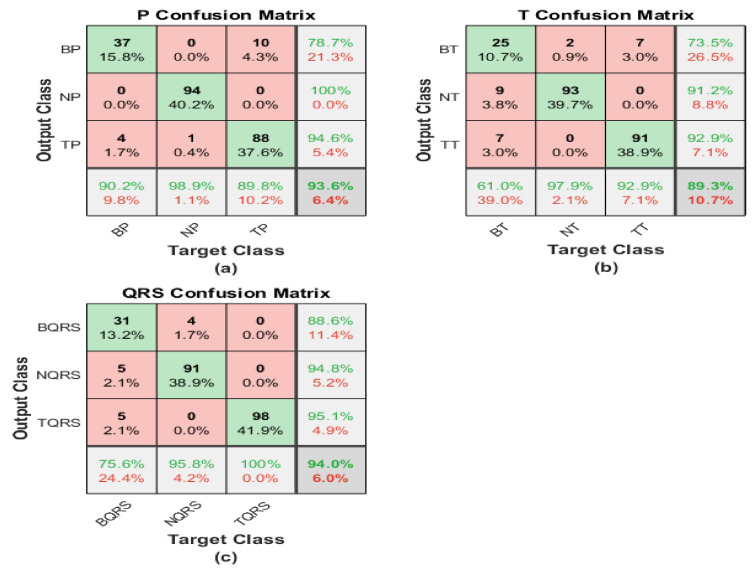
Confusion matrices of ECG irisgram using ShuffleNet: (**a**) P-waves irisgram; (**b**) T-waves irisgram; (**c**) QRS-waves irisgram.

The first confusion matrix in Figure 11a represents the P-waves irisgram images. The sensitivity of bradycardia is 90.2%, where 37 cases are classified correctly from 41. The precision of bradycardia is 78.7%. Meanwhile, 94 segments were distinguished from 95 p-waves for normal subjects, with the highest sensitivity of 98.9% and a best positive predictive value reaching 100%. A true positive rate of tachycardia is 89.8%. On the other hand, its precision is the lowest because 14 cases were misrecognized as tachycardia. The overall accuracy is 93.6% for all classes.

The second confusion matrix represents the outputs of T-waves representation. The results are not adequate. Nine segments are misclassified as healthy, and seven cases of bradycardia are misclassified as tachycardia. Therefore, the sensitivity is too low for bradycardia cases at 61%. However, nine cases from other classes are misrecognized as bradycardia by the worst precision of 73.5%. The accomplishment of ShuffleNet is better than normal class discrimination. Seven samples are misclassified as bradycardia, and zero samples as tachycardia, with high sensitivity of 92.9%, and a misclassification rate of 7.1%. There are seven classes of bradycardia classified as normal for discrimination between tachycardia and normal classes, and the precision does not exceed 92.5%. Tachycardia’s sensitivity reaches 95.9%, and the misclassification rate is 4.1%. Moreover, 19 cases of bradycardia cases are classified as tachycardia. Therefore, the precision was reduced to 82.2%. The overall accuracy is the lowest, and it does not exceed 83.3%

For the QRS waves irisgram, results show an improvement in discrimination between three classes in terms of sensitivity, accuracy, and precision. In the bradycardia class, 31 cases are classified correctly from 41, and four misclassified samples as bradycardia. That is why the precision reduced to 88.6% from what it was in the P waves case. Recall and PPV are the highest for normal ECG segments by almost 94.8% for both performance evaluation terms. Tachycardia obtains a high level of precision using the QRS-waves, in which just five segments from all the data were misclassified as tachycardia, and the highest sensitivity reaches 100%. The overall accuracy of the proposed approach regarding QRS-waves irisgram is 94.0%.

### 4.2. Scalogram Representation

#### 4.2.1. ResNet

The scalogram image recognition is performed utilizing pre-trained ResNet101 architecture, and the following matrices represent its performance. The first matrix describes the accomplishment of the scalogram of the P-waves, while the second one shows the capability level of the scalogram of the T-waves. On the other hand, the third one demonstrates the performance of the scalogram of the QRS-waves.

**Figure 12:**
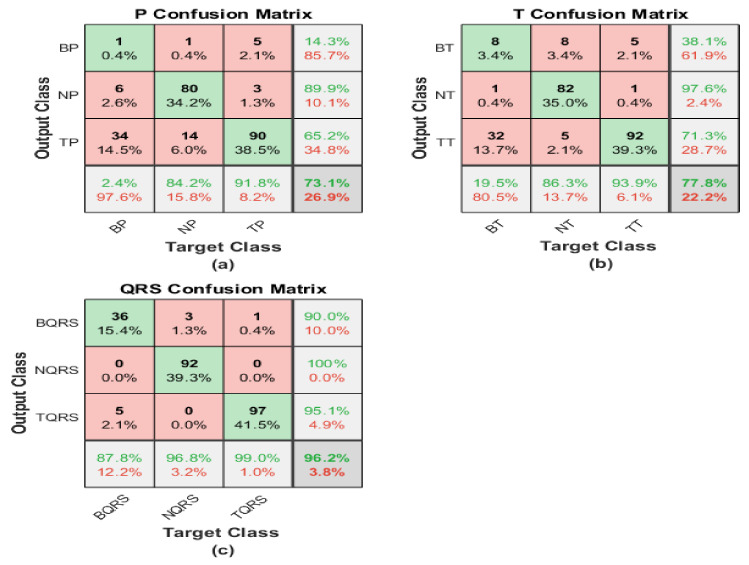
Confusion matrices of ECG SGs using ResNet101: (**a**) P-waves SG; (**b**) T-waves SG; (**c**) QRS-waves SG.

The first confusion matrix in Figure 12a indicates P-waves scalogram images. The sensitivity of bradycardia is 85.4%, where 35 cases are discriminated correctly from 41. The precision of bradycardia is almost moderate by 87.5%. While 93 segments were distinguished from 95 P-waves for healthy subjects, with the highest sensitivity of 97.9% and a best positive predictive value reaching 100%. The true positive rate of tachycardia is 94.9%. The precision is 92.1% because 14 cases were misclassified as tachycardia. The overall accuracy is 94.4% for all classes.

The second confusion matrix describes the output of T-waves representation. The results are better than the P-waves and QRS-waves scalogram. Two segments are misclassified as healthy, and no cases of bradycardia are misclassified as tachycardia. Therefore, the sensitivity is too high for bradycardia cases at 95.1%. However, two cases from other classes are misrecognized as bradycardia by the highest precision of 95.1%. The performance of ShuffleNet is better than normal class discrimination. Two samples are misrecognized as bradycardia, and zero samples as tachycardia, with high sensitivity of 97.9%, and a misclassification rate of 2.1%. For discrimination between tachycardia and normal classes, there are no misclassification segments with the highest precision reaches to 100%. Tachycardia’s sensitivity is the best by 100%. Moreover, only two cases of bradycardia cases are misclassified as tachycardia. Therefore, the precision is high at 98%. The overall accuracy is the highest and reaches 98.3%.

QRS-waves SG is presented in the third confusion matrix. In the bradycardia class, 18 cases are classified correctly from 41, and 19 segments are misclassified as bradycardia. That is why the precision is too low reaching 48.6% from what it was in the P-waves case. Recall and PPV are the highest for normal ECG segments by almost 92.8% for both performance evaluation terms. Tachycardia obtains an acceptable level of precision using QRS-waves, in which 18 segments from the whole data were misclassified as tachycardia, while the sensitivity reaches 83.7%%. The overall accuracy of the proposed approach regarding the QRS-waves SG is 81.0%.

#### 4.2.2. ShuffleNet

The scalogram image recognition is executed using a pre-trained ShuffleNet structure, and the corresponding matrices illustrate its performance. The first matrix represents the performance of the scalogram of the P-waves, while the second one shows the capability level of the scalogram of the T-waves. On the other hand, the last confusion demonstrates the performance of the scalogram of the QRS waves.

**Figure 13:**
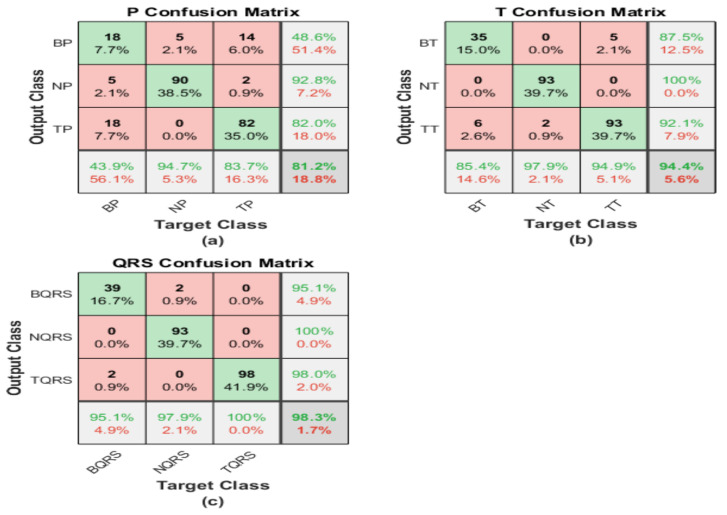
Confusion matrices of ECG SGs using ShuffleNet: (**a**) P-waves SG; (**b**) T-waves SG; (**c**) QRS-waves SG.

The first confusion matrix in Figure 13a indicates P-waves scalogram images. The sensitivity of bradycardia is 2.4%, where just one case is discriminated correctly from 41. The precision of bradycardia is too low at 14.3%. While 80 segments were distinguished from 95 P-waves for healthy subjects, with moderate sensitivity of 84.2% and a positive predictive value reaching 89.9%. A true positive rate of tachycardia is 91.8%. The precision is 65.2%, because eight cases were misclassified as tachycardia. The overall accuracy is 73.1% for all classes.

The second confusion matrix describes the outputs of T-waves representation. The results are almost like a P-waves SG. Eight segments are misclassified as healthy and five cases of bradycardia are misclassified as tachycardia. Therefore, the sensitivity is too low for bradycardia cases at 80.5%. However, 13 cases from other classes are misrecognized as bradycardia by the lowest precision of 38.1%. The performance of ShuffleNet is better than normal class discrimination. Eight samples are misrecognized as bradycardia and five samples as tachycardia, with moderate sensitivity of 86.3% and a misclassification rate of 13.7%. For discrimination between tachycardia and normal classes, there are five misclassification segments and 32 segments misclassified for bradycardia with a low precision reach of 71.1%. Tachycardia’s sensitivity is the best at 93.9%. Moreover, only five cases of bradycardia cases are misclassified as tachycardia. Therefore, the precision is high at 98%. The overall accuracy is the highest and reaches 77.6%. Table 2, Table 3, Table 4 and Table 5 summarize the obtained results. Exploiting the scalogram representation of T-waves and the pre-trained ResNet101 yields a high accuracy of 98.3%.

QRS-waves SG is presented in the third confusion matrix. In the bradycardia class, 36 cases are classified correctly from 41, and 4 segments are misclassified as bradycardia. That is why the precision is almost high reaching 90.0% from what it was in the P-wave case. Recall and PPV are the highest for normal ECG segments by almost 96.8% and 100%, respectively. Tachycardia obtains the best level of precision using the QRS-waves, in which just one segment from the whole data was misclassified as tachycardia, while the sensitivity is the best too, reaching 99.0%. The overall accuracy of the proposed approach regarding the QRS-waves SG is 96.2%.

**Figure 14:**
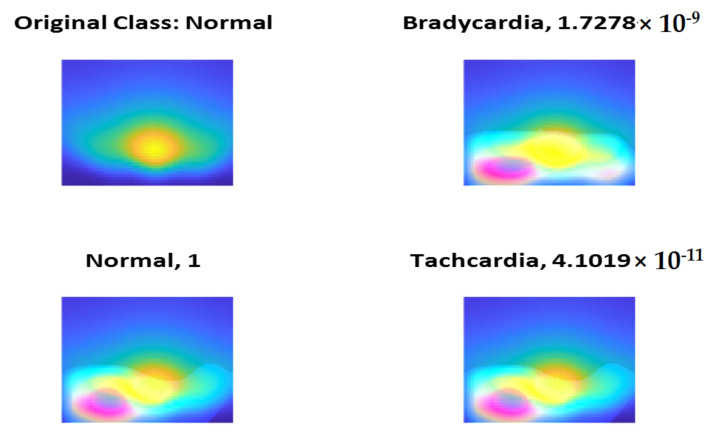
Normal Class CAM using ShuffleNet and Scalogram.

**Figure 15:**
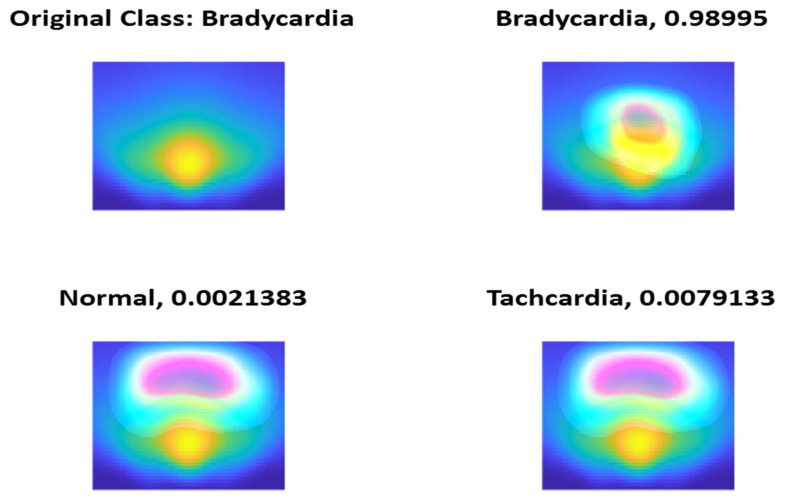
Bradycardia Class CAM using ShuffleNet and Scalogram.

**Figure 16:**
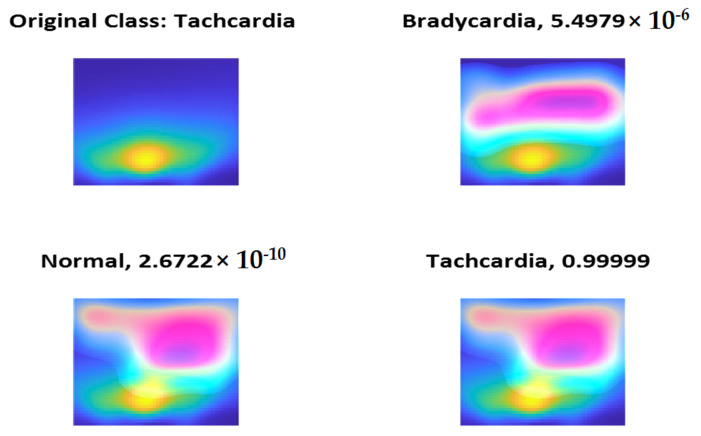
Tachycardia Class CAM using ShuffleNet and Scalogram.

To check the validity of features extracted using the models, we check the class activation maps (CAMs) and we find that all the trained models have selected the most significant regions of the scalogram and irisgram. Figure 14, Figure 15 and Figure 16 show a sample if CAM using ShuffleNet using the last ReLU layer and scalogram for the three classes Normal, Bradycardia, and Tachycardia, respectively.

### 4.3. K-Fold Results

**Figure 17:**
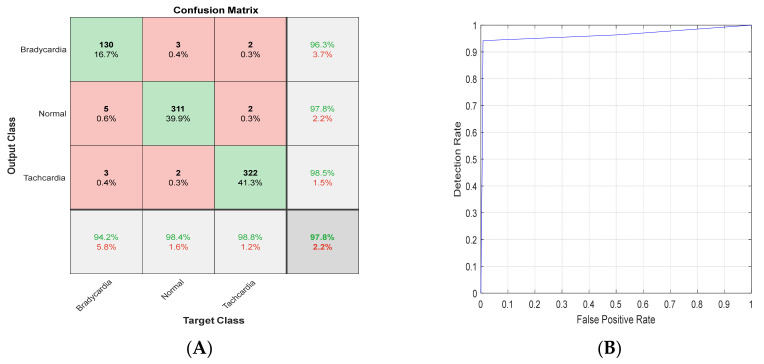
(**A**) Confusion matrix and (**B**) ROC for ResNet101 with Scalogram T-waves.

**Figure 18:**
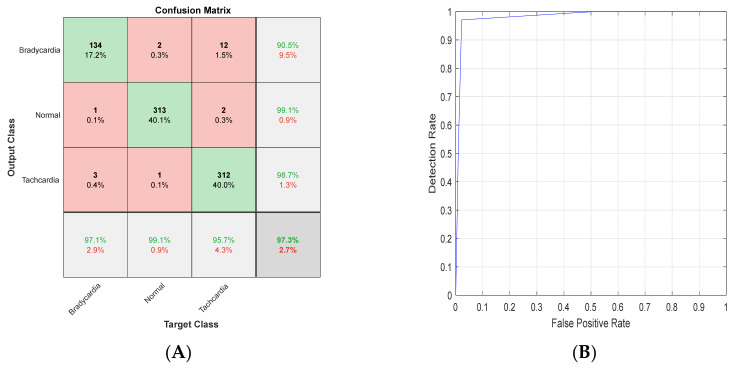
(**A**) Confusion matrix and (**B**) ROC for ShuffleNet with Scalogram QRS-waves.

To ensure the validity of the proposed methodology, the datasets’ evaluation was performed using a 5 K-fold technique. These techniques were applied on the highest two results gained using each ShuffleNet and ResNet101. The overall confusion matrix and ROC of ResNet101 with Scalogram T-waves are shown in Figure 17. The overall confusion matrix and ROC of ResNet101 with Scalogram T-waves are shown in Figure 18.

Then, the performance results of 5 K-fold using the two scenarios are shown in Table 6. Using these results, we can conclude that the performance of the proposed model is stable over different sets of training and testing, which make it robust.

## 5. Conclusions

This paper introduced a comparison between different ECG waves (P-QRS-T) spectrum representations (iris-spectrogram and scalogram) and two widely used CNN architectures (ResNet101 and ShuffleNet) for classifying three main heart rhythms (Normal, Tachycardia, and Bradycardia). The paper mainly focused on how different spectrum representations of other ECG beat waves combined with one of the two used CNN architectures perform on the classification of arrhythmias to ensure their ability to be applied later using embedded systems. The proposed methodology addressed the main concern of providing high performance with the lowest computational complexity on preprocessing, feature extraction, and classification for classifying ECG beats arrhythmias. The suggested combination methods achieved, in general, high-performance rates with generated images from these spectrums and were fed to CNN architectures. The main advantages of the proposed system are the ability to employ the methodology in embedded systems in the future. The disadvantages are the limited number of records on the used dataset, where a larger dataset is required for further evaluation of the method, yet the current results are very promising.

## Figures and Tables

**Table 1:** The amplitudes and frequencies of ECG waves.

ECG Waves	Amplitude	Frequency
P-Wave	0.25 mV	5–30 Hz
QRS-Complex	The amplitude for the largest wave R is 1.6 mV	8–50 Hz
T-Wave	0.1–0.5 mV	0–10 Hz

**Table 2:** Results of Irisgram and ResNet101.

	P-Wave		QRS-Wave		T-Wave	
	Sensitivity	Precision	Sensitivity	Precision	Sensitivity	Precision
Bradycardia	85.40%	94%	90%	86%	39%	57.10%
Normal	91.60%	98.90%	96.80%	96.80%	90.50%	92.50%
Tachycardia	96.90%	87.20%	94.90%	96.40%	95.90%	83.20%
	Accuracy = 92.7%		**Accuracy = 94.9%**		Accuracy = 83.8%	

**Table 3:** Results of Irisgram and ShuffleNet.

	P-Wave		QRS-Wave		T-Wave	
	Sensitivity	Precision	Sensitivity	Precision	Sensitivity	Precision
Bradycardia	90.20%	79%	76%	89%	61%	73.50%
Normal	98.90%	100.00%	95.80%	94.80%	97.90%	91.20%
Tachycardia	89.80%	94.60%	100.00%	95.10%	92.90%	92.90%
	Accuracy = 93.6		**Accuracy = 94**		Accuracy = 89.3	

**Table 4:** Results of Scalogram and ResNet101.

	P-Wave		QRS-Wave		T-Wave	
	Sensitivity	Precision	Sensitivity	Precision	Sensitivity	Precision
Bradycardia	85.40%	88%	44%	49%	95%	95.10%
Normal	97.90%	100.00%	94.70%	92.80%	97.90%	100.00%
Tachycardia	94.90%	92.10%	83.70%	82.00%	100.00%	96.00%
	Accuracy = 94.4%		Accuracy = 81.2%		**Accuracy = 98.3%**	

**Table 5:** Results of Scalogram and ShuffleNet.

	P-Wave		QRS-Wave		T-Wave	
	Sensitivity	Precision	Sensitivity	Precision	Sensitivity	Precision
Bradycardia	2.40%	14%	88%	90%	20%	38.10%
Normal	84.20%	84.90%	96.80%	100.00%	86.50%	97.60%
Tachycardia	91.80%	65.20%	99.00%	95.10%	93.90%	71.30%
	Accuracy = 73.1%		**Accuracy = 96.2%**		Accuracy = 77.8%	

**Table 6:** Results of 5 K-fold using two scenarios.

	T-Wave with ResNet101	QRS-Wave with ShuffleNet
Sensitivity	97.13 ± 0.95%	97.29 ± 1.30%
Precision	97.52 ± 0.23%	96.11 ± 0.17%
Accuracy	97.82 ± 0.65%	97.31 ± 0.50%

## Data Availability

Data Availability from the PhysioNet PhysioBank archive: ECG-ID set is used for normal ECG type, and the Challenge 2015 Training Sets is used for tachycardia and bradycardia types. “ECG-ID Database v1.0.0, www.physionet.org/content/ecgiddb/1.0.0”, accessed on 19 November 2022. and “PhysioNet/CinC Challenge 2015: Training Sets, archive.physionet.org/physiobank/database/challenge/2015”, accessed on 19 November 2022.
